# Sinus rhythm maintenance in persistent atrial fibrillation: 12-lead ECG multiscale entropy characterization

**DOI:** 10.1007/s11517-025-03449-0

**Published:** 2025-10-10

**Authors:** Eva M. Cirugeda, Eva Plancha, Víctor M. Hidalgo, Sofía Calero, José J. Rieta, Raúl Alcaraz

**Affiliations:** 1 Department of Signal Processing and Communications, Universidad Rey Juan Carlos, Fuenlabrada, Spain; 2 Department of Cardiology, Salut Xativa-Ontinyent, Valencia, Spain; 3 Department of Cardiac Arrhythmia, Hospital Universitario de Albacete, Albacete, Spain; 4 BioMIT.org, Department of Electronic Engineering, Universitat Politècnica de Valencia, Gandía, Spain; 5 Research Group in Electronic, Biomedical and Telecommunication Engineering, Universidad de Castilla-La Mancha, Cuenca, Spain

**Keywords:** Atrial fibrillation, Electrical cardioversion, Multiscale entropy, Sample entropy, 12-lead ECG

## Abstract

**Abstract:**

Persistent atrial fibrillation is the most common sustained cardiac arrhythmia, frequently linked with increased mortality and morbidity. Electrical cardioversion (ECV) remains the gold standard for sinus rhythm (SR) restoration, even though presenting potential adverse effects and a high relapsing rate. Predicting ECV outcome from the 12-lead ECG could reduce healthcare costs while preventing complications in patients unlikely to maintain SR. To this end, atrial activity (AA) organization has been traditionally evaluated through the amplitude and dominant frequency of the fibrillatory waves at lead II. However, physiological systems are known to exhibit complex dynamics across multiple time-scales, making multiscale (MSE) entropy measures a more suitable tool, as they can incorporate relevant information that may have been previously overlooked. Here, the predictive power of different MSE-based indices for the ECV outcome in 58 patients is evaluated. AA was estimated using a QT segment cancellation algorithm. Patients were classified based on SR maintenance after a 30-day follow-up. Results show that traditionally used indices report the highest predictive rate over the limb leads (79%). However, they are outperformed by Refined MSE over precordial leads (87%). Moreover, when considering statistical modeling techniques such as support vector machines, the prediction accuracy is increased (98%). In conclusion, MSE-based indices computed from precordial leads can robustly predict ECV outcome with higher accuracy than traditional approaches.

**Graphic abstract:**

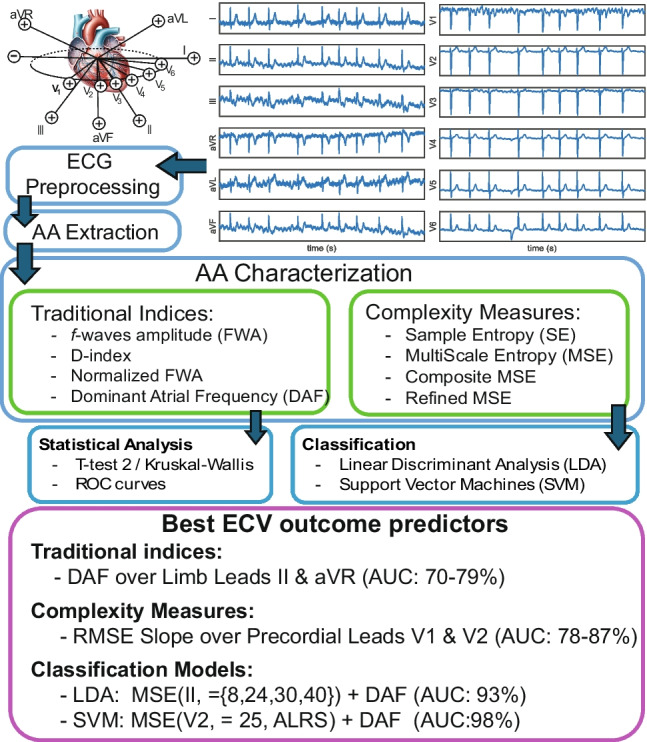

## Introduction

Atrial fibrillation (AF) is the most common sustained cardiac arrhythmia encountered in clinical practice and is increasing in prevalence as the population ages. AF is often associated with structural heart disease, an increased long-term risk of stroke, heart failure, and all-cause mortality, resulting in healthcare cost increase [1, 2]. AF is a supraventricular tachyarrhythmia characterized by uncoordinated atrial activation with consequent deterioration of atrial mechanical function. On the surface electrocardiogram (ECG), AF is described as the replacement of consistent P waves by rapid oscillations termed fibrillatory (*f*-) waves that vary in size, shape, and timing and are often associated with an irregular and frequently rapid ventricular response (RR intervals)  [1, 2].

The mechanisms causing and sustaining AF are multifactorial. AF occurs generally when structural and/or electrophysiological abnormalities alter atrial tissue to promote abnormal impulse formation and/or propagation. Different theories try to explain AF maintenance; one of them assumes multiple independent reentrant wavelets associated with heterogeneous conduction and refractoriness [2]. According to this idea, different approaches have been proposed to characterize AF organization either by clinical [3–6] or mathematical derived indices from both, invasive atrial recordings [4, 7, 8] and surface ECG [4, 7, 9–12]. From a clinical point of view, indices derived from the surface ECG result of great interest as they can be easily obtained while the risks derived from invasive procedures could be avoided [9].

AF can be classified into paroxysmal or persistent according to its temporal evolution. In paroxysmal AF, termination occurs spontaneously, while persistent AF is sustained over time and pharmacological or electrical cardioversion (ECV) is needed for its termination [1]. When pharmacological cardioversion (PCV) is not effective, patients are indicated to ECV. ECV involves an electrical shock synchronized with the intrinsic activity of the heart. This ensures that electrical stimulation does not occur during the vulnerable phase of the cardiac cycle. Successful cardioversion of AF depends on the nature of the underlying heart disease and the current density delivered to the atrial myocardium [1, 2]. Even though ECV is a low-cost, effective, and simple technique for AF termination, its effectiveness in time is limited as nearly 80% of the patients relapse to AF within a year. ECV is also considered to be the cause of several side effects such as thrombolytic events, ventricular and supraventricular arrhythmias, bradycardia, or sinus arrest [1]. Thus, predicting ECV outcome is of great interest; in this way, the healthcare costs associated with ECV procedures that fail to result in sustained sinus rhythm (SR) after 1-month follow-up could be avoided, and on the other hand, patients with low probability of SR maintenance could preserve their clinical status preventing the development of any other derived pathology.

ECV outcome prediction is performed by means of atrial activity (AA) regularity characterization from the surface ECG. To this end, several indices have been proposed in the literature [4, 5, 7–11], in both time and frequency domains. The most commonly used time-domain AA characteristics are the *f*-waves amplitude (FWA), power (FWP) [13], and the atrial cycle length (ACL) [7] along with the recently introduced complexity measures [8, 10] or topological methods [11]. In the frequency domain, the dominant atrial frequency (DAF) [7, 14] directly related to the ACL, the harmonic decay [3], the organization index [15], or regularity indices [16] are the most commonly used AA characteristics for AF regularity characterization. Complexity measures are a family of statistics that measure the regularity within a time-series, first introduced in physiological signal processing by Pincus [17] and Richmann [18] with reasonably good results. Particularly, sample entropy (SE) has been widely used for AA characterization and ECV outcome prediction [13, 19, 20]. Even though providing good results in predicting SR maintenance, SE evaluates AA on a single time-scale. However, physiological systems exhibit dynamical variations across multiple time and frequency scales, so it could be hypothesized that considering multiscale entropy (MSE) [21–24] could more accurately predict ECV outcome as it evaluates AA regularity at different time-scales.

Another lack in predicting ECV outcome is that generally only the precordial lead V1 is considered for this task. This lead selection is based on the site of recording due to its proximity to the right atrium [7] and is considered to present the largest *f*-waves amplitude compared to ventricular signals [9, 25]. ECG surface recordings in ambulatory environments usually comprise the 12-lead distribution; selecting only one lead results in discarding information that could be relevant for ECV outcome prediction. Few works in the literature deal with AA characterization among the 12 leads. Multidimensional AA analysis is considered in [5, 15, 26–28] and characterization over other lead than V1 in [4–6, 15, 25, 27, 29]. Generally, these indices have been employed for AF temporal characterization [15], PCV outcome prediction [5], catheter ablation (CA) procedures [4, 6, 27–29], or post-cardioversion AF recurrence prediction [26], but rarely considered for ECV outcome prediction.

This work proposes a full 12-lead surface ECG AF characterization by means of standard AA characteristics and MSE complexity techniques in order to explore the feasibility of improving ECV outcome prediction.

## Materials and methods

### Study population

Patients diagnosed with persistent AF referred to the ECV procedure in Hospital Universitario de Albacete and Hospital Francesc de Borja were considered for this study. These patients were chosen during daily clinical practice. They were retrospectively classified as (a) SR maintenance or (b) AF relapse, based on patient outcomes after 30-day follow-up. These patients were under antiarrhythmic drug treatment (amiodarone, flecainide, and sotalol) before the procedure and during the whole follow-up for 30 days.

**Fig. 1:**
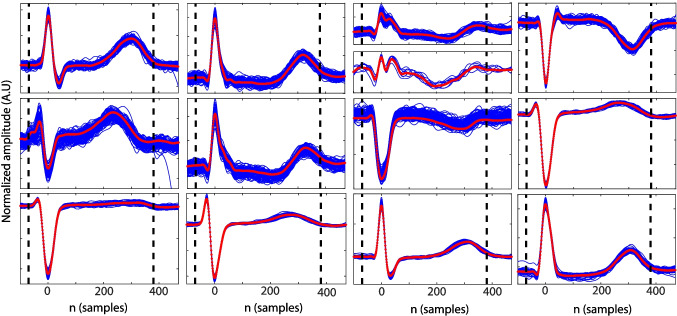
From top left to bottom right, the 12-lead ECG QT segment beat morphologies. The median QT template is drawn in red, and the QT segment duration is delimited by vertical black dashed lines

A standard 12-lead ECG was continuously recorded during the entire ECV procedure with a sampling rate of 1024 Hz and 16-bit resolution. For the application of synchronized monophasic shocks, one paddle was placed firmly in the second intercostal space on the right side parasternally, and the other paddle was placed in a left-sided lateral position along the midaxillary line. A maximum of four electrical shocks were considered, whose energy followed the increasing sequence of 200, 300, 360, and 360 J [13, 20].

**Table 1:** Clinical characteristics and measured parameters in the population under study for SR maintenance and AF relapse groups

Parameters	SR	AF
No. Patients	27	31
Men / Women	15/12	18/13
Age	63±12.43	66±12.51
Underlying heart disease	9	10
Left atrial diameter (mm)	47.32± 4.76	44.72 ± 7.32

The selection criteria were based on, first, that ECV should have been successful after the first single shock; otherwise, the patient was discarded from the study. Second, all 12-lead ECGs must be interpretable and available for analysis; if any of the 12-lead ECG recordings had unavoidable external interference or recording errors, i.e., saturated signals and electrode dispatched, patients were also discarded from the study. And last, the 12-lead ECG records should present a duration of at least 1.5 min prior to the first electrical shock. Finally, 58 records were available for a multiple lead AA characterization; clinical details can be found in Table 1.

### Electrocardiogram preprocessing and atrial activity extraction

Each of the 12-lead was independently processed for baseline wander (BW), powerline interference (PWL), and high frequency (HF) noise removal. BW was estimated by backward-forward IIR filtering using a 3rd-order Butterworth low-pass filter. A notch filter with 50 Hz central frequency and 4 Hz bandwidth was considered for PWL removal, and HF noise above 40 Hz was reduced [30]. The *jqrs* detector [31] was used for R-peak location; input parameters were set according to signal characteristics. Beats were classified based on their QRS morphology by template matching (see Fig. 1).

**Fig. 2:**
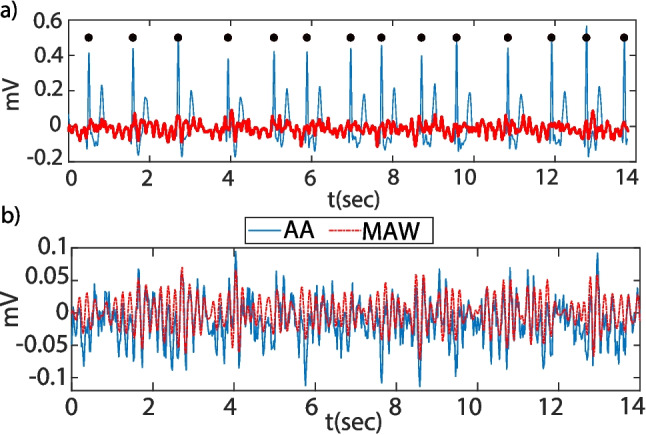
**a** Preprocessed ECG (blue) vs. extracted AA (red). The black dots indicate QRS location. **b** Estimated MAW (red dash-dot) vs. derived AA signal (blue)

An average QT cancellation algorithm was used for AA extraction [32]. QT segment length was set to the minimal duration between 470 ms (normal QT segment) or 90% of the RR intervals. The QT removal was performed sequentially for each beat class found in the signal (see Fig. 1). Beat classes were defined based on QRS complex morphology, using a clustering-based algorithm [30]. AA was then high-pass filtered at 3 Hz for remaining T wave residual removal (see Fig. 2).

### Atrial activity characterization

Consider *aa*(*n*) with *n*=0:*N*-1, the discrete AA signal, the absolute FWA is estimated by two different methodologies. First, FWA can be derived from the absolute power of the AA signal [12]:

1 $$\begin{aligned} FWA = \sqrt{\frac{1}{N}\sum _{n=0}^{N-1}{|aa(n)|^2}} \end{aligned}$$

Second, the index *D* allows summarizing the information from *f*-waves over the whole recording and its peak-to-peak amplitude pattern. This index is computed from the envelope differences [28]:

2 $$\begin{aligned} D(aa) =\frac{1}{N}\sum _{n=0}^{N-1}{\Vert e_{MAX}(n) - e_{MIN}(n)\Vert ^2} \end{aligned}$$

where e$_{MIN}$ and e$_{MAX}$ denote de minima and maxima envelopes, respectively, obtained by interpolating between the estimated local maxima or minima of aa (*n*).

The normalized FWA (FWAn) is estimated by normalizing the FWA by the mean amplitude of the QRS complexes, according to [12]:

3 $$\begin{aligned} FWAn = \frac{\sqrt{\frac{1}{N}\sum _{n=0}^{N-1}{|aa(n)|^2}}}{\sqrt{\frac{1}{N_b}\sum _{i=0}^{N_b-1}{|x(i)|^2}}} \end{aligned}$$

where x(*n*) denotes the ECG signal, *i* indices the R-peak detection and $N_b$ denote the number of beats.

The DAF is defined as the frequency where the maxima of the power spectral density (PSD) of aa(*n*) between 3 and 12 Hz is found, according to [12]:

4 $$\begin{aligned} DAF = \texttt {arg}{\texttt {max}}\{\overline{PSD}(f_i)\}, f_i\in [3, 12]Hz \end{aligned}$$

The PSD is computed over signal segments of 6 s in duration and 2 s overlap between adjacent segments, using a Hamming window. For each segment *i*, a PSD$_i$ is computed. Cross correlation coefficients (CCs) are then calculated between each PSD and all other PSDs. The PSD with the highest number of pairwise CCs greater than 0.7 is selected as the dominant pattern. All PSDs with a CC > 0.7 relative to this template are averaged to obtain the final mean PSD used for DAF estimation, hence avoiding signal segments with large amounts of noise, QT residue, or any other external interference.

AA organization is also characterized in the time domain by sample entropy (SE). SE has been widely used for AA characterization previously, i.e., [3, 4, 13, 19, 20, 33, 34]. Complexity measures are a family of statistics that evaluate the self-similarity within a nonstationary time-series; it is widely known that lower complexity values are related to more regular or organized time-series with higher self-similarity [18]. In order to perform a robust and reliable characterization of the AA, the main atrial wave (MAW) or main *f*-waves component needs to be estimated. The MAW can be considered the signal associated with the fundamental atrial waveform, its wavelength being the inverse of the AA main frequency [7]. The MAW is estimated by bandpass filtering the AA signal by means of a 9th-order IIR Chebyshev type 2 filter centered in the DAF and 5 Hz bandwidth, ensuring an attenuation of 20dB in the stop band, and an excerpt of the estimated MAW compared to the original derived AA activity is presented in Fig. 2.

SE is defined as the logarithmic likelihood ratio that two sequences that match for *m* points will do it as for $m+1$ points. Its computation relies on two main parameters, the embedded dimension (*m*) and the dissimilarity threshold (*r*). Here, *m*=2 and *r*=0.2$\sigma$ are considered following the recommendations of [18] for physiological time-series, where $\sigma$ stands for the standard deviation of the signal being analyzed. Thus, considering *ff*(*n*) with *n*=0:*N*-1 to be the MAW signal, SE is estimated in the following [18]:

1. Compute epochs of length *m*:
  5 $$\begin{aligned} v_{m}(n) = \left\{ ff(n+i) : \hspace{0.25cm} 0 \le i \le m - 1 \right\} \end{aligned}$$
2. Calculate the distance between every possible pair of epochs:
  6 $$\begin{aligned} {\begin{matrix} d_{jk}(m) = \max \left\{ |v_m(j) - v_m(k)| \right\} \end{matrix}} \end{aligned}$$
3. Estimate the number of matches of length *m*:
  7 $$\begin{aligned} B_k^m(r) =\frac{1}{N-m-1}\sum _{\underset{\scriptstyle j \ne k}{\scriptstyle j=1}}^{N-m}{(d_{jk}(m)<r)} \end{aligned}$$
4. Compute the probability that two epoch of length *m* will match for a distance *r*:
  8 $$\begin{aligned} B^m(r) = \frac{1}{N-m}\sum _{i=1}^{N-m}{B_i^m(r)} \end{aligned}$$
5. Repeat steps 1–5, increasing the epoch length in one sample, $m+1$
6. Estimate SE, as follows:
  9 $$\begin{aligned} SE(ff,m,r,N) = - \ln \frac{B^{m+1}(r)}{B^m(r)} \end{aligned}$$

Generally, time-series derived from complex systems are likely to present structures across multiple spatio-temporal scales, something SE does not account for. Costa et al. proposed multiscale entropy analysis (MSE) in order to account for time-series complexity at multiple time-scales [21, 22] and provide a more robust entropy characterization.

MSE modifies the SE algorithm in the way that the subvectors defined in Eq. 5 are estimated over a coarse-grained version of the MAW signal at each time-scale $\tau$. The coarse-grained series for time-scale $\tau$, $ff^{\tau }(n)$ is computed according to the following:

10 $$\begin{aligned} ff^{\tau }(j) = \frac{1}{\tau }\sum _{i=(j-1)\tau }^{j\tau }{\hspace{-0.3cm} ff(i)} \hspace{0.5cm} 1 \le \ j \le \frac{N}{\tau } \end{aligned}$$

SE is then computed over each of the coarse-grained series. Here, $\tau _{max}$ = 40 so as to provide enough resolution and robustness in the measure at large temporal scales. MSE is defined as follows:

11 $$\begin{aligned} MSE(ff,\tau ,m,r,N) = SE(ff^{\tau },m,r,N/\tau ) \end{aligned}$$

MSE lacks an increase in the variance of the estimated entropies at large time-scales; this increase in variance is generally due to signal length restrictions [23]. To overcome this limitation, Wu et al. defined composite MSE (CMSE) [23], where MSE is not computed over a unique coarse-grained series, but estimated as the mean of the SE entropies computed over the different *k* coarse-grained series that can be derived from the MAW signal:

12 $$\begin{aligned} {\begin{matrix} ff_{k}^{\tau }(j) = \frac{1}{\tau }\sum _{i=(j-1)\tau +k}^{{j\tau +k -1}}{\hspace{-0.5cm} ff(i)}, \\ 1\le j\le \frac{N}{\tau } \hspace{0.5 cm} 1 \le k \le \tau \end{matrix}} \end{aligned}$$

13 $$\begin{aligned} CMSE(ff,\tau ,m,r,N) = \frac{1}{\tau }\sum _{k=1}^{\tau }{SE(ff^{\tau }_{k},m,r,\frac{N}{\tau })} \end{aligned}$$

MSE-based entropies present a complexity increase associated with the coarse-graining procedure. To counteract this limitation and avoid the uncontrolled effects on complexity assessment due to the suboptimal fast time-scale elimination procedure, Valencia et al. defined the refined MSE (RMSE) [24]. This entropy measure presents two major differences: (i) the coarse-grained series computation procedure and (ii) the dissimilarity threshold. The coarse-grained series for RMSE is estimated by filtering *ff*(*n*) with a 6th-order LP Butterworth filter and normalized cut-off frequency $f_c = 0.5/\tau$ applying backward-forward IIR filtering to perform a zero-phase filtering [24, 30] and subsequently downsampling it by the scale factor $\tau$. The dissimilarity threshold is set as a percentage of the standard deviation of the coarse-grained series, rather than the original time-series, as it is in SE, MSE, and CMSE.

MSE implies downsampling of the time-series, taking into account the sampling frequency of the MAW signal, the maximum DAF that can be found in the MAW signal, and the filter bandwidth, and applying the Nyquist criteria, time-scales larger than $\tau$ = F$_s$/(2*DAF$_{max}$) = 34.48, should be considered with carefulness.

According to standard MSE behavior and the observed results, three areas have been defined: (i) short-range time-scales (SRS) ranging from $\tau$ = 1 to 6, (ii) medium-range time-scales (MRS) accounting for complexity at time-scales $\tau$=6:20, and (iii) long-range time-scales (LRS), starting at 20 up to 40. Thus, MSE-based indices have been characterized by the area under the MSE curve ($\mathcal {A}$) [35] and the slope ($\mathcal {S}$) [36] over the stated regions. The $\mathcal {A}$ represents the global complexity of the signal and is estimated as the integral over each time-scale range. The $\mathcal {S}$ quantifies how rapidly the complexity changes with time-scale and is thought to be related to the severity of the illness condition. It is computed by fitting a first-degree polynomial using ordinary least squares regression.

### Statistical analysis

The statistical distribution of the estimated parameters was evaluated by the Kolmogorov-Smirnov (KS) hypothesis test [37]. If data were well modeled by a normal distribution, the two-sample *t*-test (T-Test2) [38] was considered; otherwise, the Wilcoxon rank sum test [39] was applied. These tests evaluate the statistical significance that the two data groups considered, SR maintenance and AF relapse, come from similar distributions with equal mean or median values, respectively. In both tests, the null hypothesis is rejected with a significance level of 5$\%$.

**Fig. 3:**
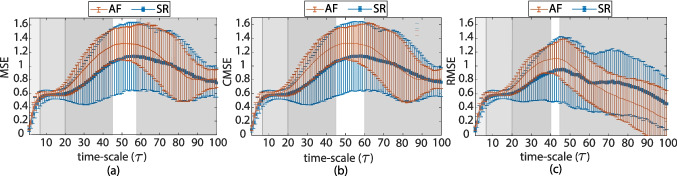
Median and interquartile ranges at each time-scale $\tau$ for **a** MSE, **b** CMSE, and **c** RMSE computed over the 12-lead ECG for SR maintenance in blue and AF relapse groups in red. Gray shadowed regions represent $\tau$ ranges where a similar behavior is found

The ECV outcome predictive power of the different parameters was studied by means of the receiver operating characteristic (ROC) curves. This plot provides information on how each parameter can be used as a classifier by computing the true positive (sensitivity) and true negative (specificity). The area under the curve (AUC) can be considered an aggregate measure of performance of the variable of interest across all possible classification thresholds. Hence, the AUC is an effective way to summarize the overall diagnostic accuracy [40]. To overcome the possible inaccuracies derived from low sample size, bootstrapping has been considered for AUC computation, considering 1000 iterations and resampling with replacement following [41, 42]. In the present work, sensitivity (Se) was considered the proportion of patients who maintained SR after a 30-day follow-up, whereas specificity (Sp) was defined as the percentage of patients who relapsed to AF. Moreover, the optimal threshold to discern between patients was computed according to Youden’s criterion [43].

### Machine learning and classification models

Following [13, 44–46], machine learning and classification models were considered to increase the diagnostic ability with respect to that obtained by using single MSE-based indices.

Two distinct classifiers were considered: linear discriminant analysis (LDA) [47], and a Gaussian kernel support vector machine (KSVM) [48]. LDA comprises a more simple problem as it assumes linear separability between classes. In contrast, KSVM utilizes kernel functions to map the original data into a higher-dimensional space, allowing the algorithm to model non-linear decision boundaries. This enables KSVM to capture more complex relationships between features and outcomes when compared to LDA.

**Fig. 4:**
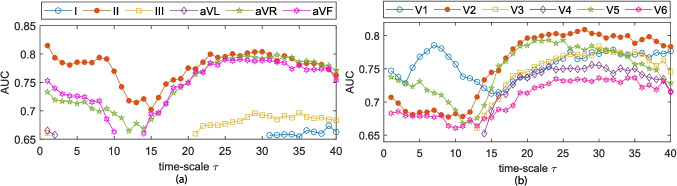
MSE classification accuracy (AUC) at different time-scales for **a** limb leads and **b** precordial leads. Only AUC values with enough statistical significance ($p<$ 0.05) are given

**Fig. 5:**
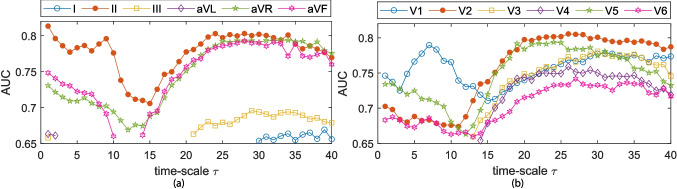
CMSE individual scale values classification accuracy (AUC) for **a** standard ECG leads and **b** precordial ECG leads. Only AUC values with enough statistical significance ($p<$ 0.05) are given

For each ECG lead, an independent classification model was developed. A sequential feature selection (SFS) algorithm was implemented to identify relevant features, considering both complexity-based measures and their combinations with traditional indices [49]. To prevent overfitting during feature selection, a ten-fold random non-stratified cross-validation was applied, using the maximization of the cross-validated AUC as the selection criterion.

After feature selection, model performance was evaluated using a bootstrap validation strategy with 1000 iterations. In each iteration, a bootstrapped set was built by random sampling with replacement, 90% of the set was considered for training, and the remaining 10% composed the out-of-bag (OOB) samples. Models were trained considering the bootstrap training sets and evaluated on the corresponding OOB sets, with AUC computed for performance assessment [41, 50].

For each classification model, the combination between the complexity approach and the lead yielding the highest validated AUC was selected.

## Results

Figure 3 depicts the median MSE value estimated from the 12-lead characterization along the different time-scales considered. Different behaviors among the MSE curves can be appreciated, which allows for the segmentation into different time regions. The SRS (1 $\le \tau \le$ 6) shows a high increase in complexity values, and in the MRS (6 $\le \tau \le$ 20), a more or less constant behavior is found, which is followed by a monotonic increase in the complexity values in the LRS (20 $\le \tau \le$ 34). The upper limit of the LRS is found to be different depending on the approach considered. The complexity increase in the LRS is higher for the AF relapse group than for the SR maintenance. From $\tau$ = 50, a monotonic decrease in the complexity is found, being more accused in the AF relapse group signals. Higher time-scales can be discarded according to the frequency distribution of the MAW as described in Sect. 2.3; particularly, MSE values for $\tau \ge$ 35 should be considered with carefulness.

MSE and CMSE display a similar behavior among all time-scales; small differences can be found in the interquartile ranges for $\tau \ge$ 40. RMSE presents higher differences, and maximum complexity values are found to be lower at the MRS and LRS for both, SR maintenance and AF relapse groups. Higher decay in complexity is found for $\tau \ge$ 45, in both groups, being more prominent for the AF relapse group.

**Fig. 6:**
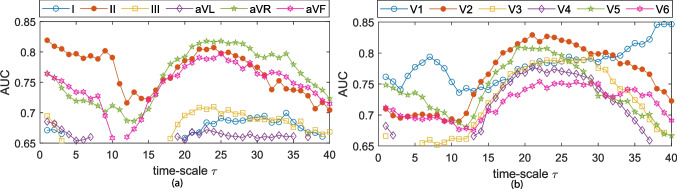
RMSE individual scale values classification accuracy (AUC) for **a** standard ECG leads and **b** precordial ECG leads. Only AUC values with enough statistical significance ($p<$ 0.05) are given

The individual index classification capabilities were evaluated by means of ROC curves; these results are given in Figs. 4, 5, and 6. Only AUC values at time-scales where statistical significance was proven ($p\le$ 0.05) are plotted. At the SRS and MRS, a general decay in AUC is observed for all leads but V1. After reaching a minima AUC around $\tau$ = 15, a generalized increase in AUC is observed for limb leads II, aVR, and aVF, and all of the precordial leads. RMSE depicts a general decay in terms of AUC for all leads but V1 in the LRS time-scales ($\tau \ge$ 25). The largest AUC values in RMSE are observed in V1 for $\tau \ge$ 38; however, as previously mentioned, $\tau \ge$ 35 have to be accounted for with carefulness. Leads II and V1 exhibit the highest AUC in the SRS and MRS time-scales for all three MSE approaches. Across the LRS time-scales, leads II and V2 are found to outperform any other lead in terms of AUC when MSE or CMSE is considered. For RMSE at LRS, lead V2 shows the higher AUC values for time-scales $\tau$ = 21:32.

MSE curves were characterized with the aggregated indices: $\mathcal {S}$ and $\mathcal {A}$ over the SRS, MRS and LRS time-scales previously defined. These metrics generally provide a better discriminant accuracy at the LRS. Table 2 provides the statistical characterization for each metric at the lead with best AUC performance for (i) the individual time-scale metrics at the best time-scale and (ii) the $\mathcal {A}$ and $\mathcal {S}$ at the time-scales regions with higher AUC.

**Table 2:** AA characterization. Indices and leads with best performance in terms of AUC

	Parameter	Lead	SR	AF	Se	Sp	AUC (%)	*p*
	FWA	II	0.051 (0.032)	0.042 (0.019)	0.704	0.581	70.44 ± 6.80	0.008
	D(aa)	aVR	0.046 (0.020)	0.036 (0.019)	0.556	0.710	63.73 ± 6.64	0.086
	FWAn	V3	0.043 (0.039)	0.028 (0.015)	0.741	0.742	77.88 ± 6.16	0.001
	DAF	II	4.883 (0.946)	5.981 (0.854)	0.806	0.741	79.07 ± 5.72	0.001
	SE	II	0.087 (0.030)	0.116 (0.032)	0.815	0.677	78.45 ± 5.86	0.001
MSE	SE($\tau$=28)	V2	0.705 (0.289)	0.903 (0.179)	0.806	0.741	80.90 ± 5.92	0.001
	$\mathcal {A}_{LRS}$	V2	16.018 (6.995)	20.102 (3.277)	0.806	0.741	79.50$~\pm$ 6.27	0.001
	$\mathcal {S}_{LRS}$	V1	0.016 (0.017)	0.026 (0.008)	0.839	0.704	78.29 ± 6.07	0.002
CMSE	SE($\tau$=26)	V2	0.667 (0.243)	0.836 (0.154)	0.806	0.741	80.54 ± 5.76	0.001
	$\mathcal {A}_{LRS}$	V2	16.002 (6.989)	20.119 (3.249)	0.806	0.741	79.82 ± 5.98	0.001
	$\mathcal {S}_{LRS}$	V1	0.016 (0.017)	0.026 (0.008)	0.836	0.704	78.29 ± 6.18	0.001
RMSE	SE($\tau$=21)	V2	0.615 (0.135)	0.721 (0.110)	0.871	0.704	82.92 ± 5.52	0.001
	$\mathcal {A}_{MRS}$	II	8.821 (0.941)	9.259 (0.225)	0.815	0.742	81.29 ± 6.06	0.001
	$\mathcal {S}_{LRS}$	V1	0.014 (0.010)	0.024 (0.004)	0.778	0.839	86.69 ± 4.63	0.001

Median values and interquartile ranges for SR maintenance and AF relapse groups, sensitivity (*Se*), specificity (*Sp*), discriminant ability (*AUC*, $\%$), and statistical significance (*p*) are given. Lowest resolution for *p* = 0.001 is considered

According to Figs. 4, 5, and 6 and Table 2, complexity metrics show better performance over precordial leads, particularly single-scale metrics best discriminant abilities estimated for lead V2 in the LRS. MSE and CMSE aggregated complexity indices, $\mathcal {A}$ and $\mathcal {S}$, best discriminant accuracy was achieved at precordial leads V1 and V2, respectively, across the LRS, along with a more homogeneous behavior. RMSE provides better accuracy results at precordial leads for single-scale indices and $\mathcal {S}$, while the $\mathcal {A}$ provides better performance at limb lead II. Among the proposed complexity approaches, RMSE provides the best trade-off between Se and Sp.

To allow comparability between the newly proposed MSE-based indices and standardly used ones for *f*-waves characterization, the latter were also independently computed among the 12 ECG leads; results are also given in Table 2. Standard amplitude indices estimate larger values for SR maintenance patients while DAF and SE estimate larger values for the AF relapse group. Standard indices provide better performance over limb leads, while MSE approaches show higher discriminating abilities over precordial leads. RMSE approach outperforms the DAF, by about 3%, even though less balanced Sp and Se values are provided.

Finally, an LDA and a KVSM model were built for each multiscale approach so as to improve the individual discriminant abilities achieved by each index. The parameters entered the SFS in terms of the individual AUC value and were selected based on the maximization of the AUC. They were combined with amplitude and/or frequency standard indices. Results are given in Table 3, for each complexity approach at the lead with the best AUC performance. Among standard parameters, DF shows to increase in model performance in 4 of the cases, while amplitude indices are only relevant in two of them. LDA’s best performance is obtained over limb lead II, while KSVM estimates higher classification accuracy among precordial lead V2 for MSE and CMSE and limb lead III for RMSE. Taking into account machine learning techniques for ECV outcome prediction outperforms single indices up to 17%.

**Table 3:** Complexity (*Cx*) models for each Cx approach (*Appr*) statistical characterization

Model	Appr	Lead	Parameters		ROC characteristics		
			Cx	Standard	Se	Sp	AUC (%)
LDA	MSE	II	$\tau$={8, 24, 30, 40}	DF	0.852	0.968	93.55 ± 5.62
	CMSE	II	$\tau$={30, 36}, $\mathcal {A}_{LRS}$		0.852	0.903	86.26 ± 7.03
	RMSE	II	$\tau$={23, 24, 28, 32, 40}	D(aa)	0.701	0.934	84.11 ± 5.30
KSVM	MSE	V2	$\tau$=25, $\mathcal {A}_{LRS}$	DF	0.926	0.936	97.85 ± 1.94
	CMSE	V2	$\tau$={25}, $\mathcal {S}_{LRS}$	DF + FWA	0.968	0.926	96.77 ± 2.10
	RMSE	III	$\tau$={24, 28}, $\mathcal {A}_{MRS}$, $\mathcal {A}_{LRS}$	DF	0.926	0.968	96.42 ± 2.30

Model, parameters of the model, and ROC curves performance metrics, sensitivity (*Se*), specificity (*Sp*), and accuracy (*AUC*)

## Discussion

### MSE-based indices

All three MSE-based curves in the SRS describe a rapid and monotonic increase in complexity values. This increase is commonly attributed to the coarse-graining process, particularly the reduction of oversampling effects [22, 24]. As noted by [18], considering too high sampling frequencies can lead to an overestimation of matches and consequently a biased complexity estimate.

In the MRS, complexity values tend to stabilize. The coarse-graining process is responsible for attenuating frequency components within 50 and 150 Hz, and the intrinsic decimation implies a reduction in the sampling frequency by the time-scale, leading to $F_s$ between 51.2 and 170.66 Hz, hence allowing for a maximal frequency representation of 85 and 25 Hz, respectively. Taking into consideration the MAW estimation process, (i) the QT interval is reduced and (ii) the AA signal is bandpass filtered around the DAF, thereby minimizing any content whose frequency lies around 20 Hz. These factors contribute to the more or less constant behavior in the entropy values. These findings are similar to those in [35], though single-channel data was considered here.

A constant monotonic increase is displayed in the LRS, reaching a maximum at a time-scale in between 40 $\le \tau \le$ 60, after which a general decay is observed. The LRS is often associated with slow time-varying processes and might be related to the probability of AF maintenance through atrial remodeling, as frequencies closer to the DAF are being analyzed [12]. This decrease in complexity is often interpreted as a consequence of information loss inherent to the coarse-graining procedure. The point at which the behavior changes is somehow related to the DAF as for the SR maintenance group, which present a lower median DAF; this point corresponded to higher time-scales than for AF relapse group, and this finding suggests that the time-scale at which this behavior changes limits somehow the interpretability of the results and could be used as a complementary index for ECV outcome prediction.

Contrary to the three regions defined here, most of the works found in the literature [21–24, 51] establish only the two initial regions. However, they only deal with time-scales below 20 along with nonuniformly adimentionally sampled sequences or time-series with lower sampling frequency and lower frequency content. Nonetheless, the works [52, 53] were found to consider three regions similar to those considered here. Bari et al. [52] defined narrower time-scale regions with respect to the ones defined here, but considering that the heart rate variability signal is characterized by frequency content below 0.5 Hz and a sampling frequency of 2 Hz is considered, a similarity between the time-scale ranges can be built. However, the aggregated entropies are not comparable as they are estimated as the mean value of the entropies at each time-scale range. Moreover, in [53], the curves obtained for alpha waves of the electroencephalogram were found to show similar complexity curves depicting the three considered regions here. Among other differences, the major similarity between alpha waves and *f*-waves is that they present similar frequency content [53]. This finding puts again in relevance the idea that larger time-scales, close to the frequency content of analysis, are able to better reflect the internal dynamics of the generating systems.

MSE generally associates higher values to AF relapse than to SR maintenance in the considered meaningful time-scale ranges. These findings agree with existing literature [13, 21] where more dynamically complex or less organized systems produce higher complexity values. AF is characterized by random atrial activations [2]; the probability of AF maintenance is related to the refractoriness of the atria [12], and then it could be hypothesized that subjects with higher probability of AF maintenance will present a broader and larger number of activations and wavefronts in the atria that could aid the triggering or maintenance of AF at different time-scales [54]. Consequently, more dynamically complex signals will be generated which will result in higher entropy values (see Fig. 3). The entropy of these signals will be better represented by MSE-based measures that can provide a more comprehensive view of the signal’s structural complexity, avoiding the inherent limitations of single-scale entropy approaches like SE, which fail to capture complex patterns that emerge across multiple time-scales, which is an intrinsic characteristic of physiological systems [22].

The largest separation between MSE values was found in the LRS, even though it presented higher interquartile ranges. The slopes in the LRS are also found to be more accused for the AF relapse group, reaching its maxima at a lower time-scale than for the SR maintenance group. This leads to the thought that the AF signal needs a higher sampling frequency for a complete representation of the spectral content, which means that higher DAFs are present, just as expected from the literature [13].

CMSE was developed to reduce the variance of the estimated SE at large time-scales [23]. Even though MSE and CMSE show a very similar behavior, a more uniform evolution in CMSE interquartile ranges was found [23]. RMSE not only modifies the way in which the coarse-grained series is computed, but also the threshold *r* is set as a function of the coarse-grained series standard deviation rather than the original signal standard deviation [24]. According to [24], an increase in RMSE would have been expected with respect to MSE values. Apparently, it does not happen here, as relatively smaller values were estimated for RMSE in the LRS. When comparing both groups at the same time-scales, RMSE estimates higher complexity values than MSE for all time-scales up to $\tau = 30$, and then RMSE shifted to lower entropy values. It is worth noticing too that the RMSE decay is more abrupt after the entropy maximum; this might be due to the coarse-graining procedure. For instance, time-scales $\tau \ge$ 40 imply a sampling frequency $F_s\le$ 25 Hz, hence allowing a maximal representation of around 12 Hz. The MAW signal frequency components are located in the range [0, 13] Hz. Then, the differences found between RMSE and CMSE/MSE curves at time-scales $\tau \ge$ 40 are mainly due to the alias that remains in the signal after the coarse-graining procedure. While CSME/MSE uses a mean-based algorithm, RMSE low-pass filters the signal previously, hence removing all frequency components causing the alias.

### Predictive accuracy and optimal lead selection

The classification performance for each considered index was independently evaluated for each ECG lead using ROC curves analysis. Accuracy was assessed through Se and Sp and AUC estimations. Optimal lead selection for discrimination between AF relapse and SR maintenance groups was based on the highest AUC values.

The best performance among traditionally used indices was obtained over limb leads for all of them but FWAn. The FWA and FWAn are higher for SR maintenance group than for AF relapse; this finding is supported by previous works, and more organized signals presented lower DAF and higher amplitudes of the *f*-waves [4, 5, 13, 54]. The newly proposed index in [28] D(aa) reported results with limited accuracy that did not provide significant differences between groups. This index is computed based on envelope differences, and results suggest that baseline AF signal amplitude cannot be fully characterized by them, thus supporting the idea that the organization in the signal recorded previously to an ECV protocol is more complex than that found in signals recorded after CA, as in [28] where a median AUC of 0.98 with sufficient statistical significance over lead V3 was obtained. This leds to the third amplitude characteristic analyzed, FWAn, which reported the best classification results in lead V3 (see Table 2) with enough statistical significance. According to [9, 12], it would have been expected that the best accuracy results were obtained in lead II or V1. This finding can possibly be related to the QT cancellation algorithm and the remaining residue in the AA signal. Figure 1 provides the QT segment morphology at every lead in the surface ECG. Lead V3 presents a high QRS complex against a nearly inexistent T wave. This finding allows to hypothesize that the residues left in the AA signal after the QT segment cancellation might be negligible when compared to any other lead in the surface ECG while still reflecting enough AA for its characterization, which suggests the importance of the QT cancellation algorithm. Moreover, lead V3, similarly to lead V2, provides an intermediate signal between both atria, thus suggesting the importance of considering space dynamics in the analysis. The estimated DAF values are higher for the AF relapse group, just as in [4, 13, 19]. Its best predictive value was found for lead II as in [5]. Finally, SE best discriminant ability was found over lead II just as in [5]. According to the findings in [6, 25], these results suggest that this index reports better discriminant abilities based on the area of generation or maintenance of AF. At single time-scales, the triggering phenomena could be more relevant than the wavefront propagation, thus suggesting that a higher number of atrial ectopic beats originate in the right atria [6, 25, 55].

MSE-derived indices predictive rates based on AUC estimates at each time-scale are given in Figs. 4, 5, and 6 for each lead. Higher AUC values for lead II among the limb leads and for lead V1 in the SRS among the precordial ones are reported. These findings are consistent with [9, 12], who state that leads II and V1 are the ones reporting higher AF activity, particularly at the SRS region. For most ECG leads, AUC values experiment a decay until approximately $\tau =$ 12, and then they begin to increase, often reaching or surpassing the values observed in leads II and V1. This increase in AUC can be directly related to a larger separation between the groups. Limb leads, particularly lead II, seem to provide better discriminant ability at the SRS, while precordial leads do it in the LRS. MSE and CMSE show similar AUC trends while in the case of RMSE, lead aVR showed up to be the outperforming one from $\tau \ge$ 15.

The overall best-performing leads for MSE-based methods were the precordial leads in the LRS, as shown in Table 2. Specifically, lead V2 yielded the highest AUC for single-scale entropy measures for all three MSE approaches. In the LRS region, the prediction rate reached AUC values higher than 80%, hence outperforming the DAF in at least 1 point up to 3 points in RMSE. These results aid to think that AF does present long-range correlations that have a higher effect on the AF relapsing processes that traditional indices cannot detect. In [54], early AF is associated with a high incidence of triggers such as atrial premature contractions (APC). This APC cannot be captured by the DAF, neither in SE, but it could have been unveiled by the use of RMSE at the optimal time-scale. Moreover, even though lead V1 is chosen among the vast majority of works as considering AF due to it proximity to right atria, i.e., [4, 10, 13, 25, 27, 45], or lead II is considered to present the higher monophasic P wave [12, 56], lead V2 is thought to provide an intermediate signal between the right (V1) and left (V5) atria, potentially offering valuable information on atrial wavefront propagation.

Furthermore, the aggregated MSE-based index $\mathcal {A}$ reported the highest AUC values at V2 for MSE and CMSE, while lead II was chosen for the RMSE approach. $\mathcal {S}$ reported optimal results at lead V1 for all three MSE-based approaches. While they do not outperform single scale measures for all three MSE approaches, the $\mathcal {S}$ of RMSE computed over lead V1 increases the discriminant ability up to 86% along with a better trade-off between Se and Sp. This suggests that considering the analysis of MSE for a range of time-scales is valuable for evaluating the regularity of the *f*-waves activity, as similar findings were obtained in [57] even though a different coarse-grained procedure based on moving average filtering was considered. These results are also supported by [35, 36, 58] where statistically significant differences were found for the LRS rather than for the SRS, even though the signals and entropy-based approaches are quite distinct. However, a deeper study over a more extensive database should be performed.

The improved predictive capacity of MSE-based metrics compared to the traditionally used indices may be explained by the capacity of MSE to characterize not only linear but also nonlinear dynamics of AA across multiple temporal scales. Traditional indices primarily capture linear or single-scale properties of the signal, which may be insufficient to reflect the changing dynamics of AA activity and the multiscale nature of atrial remodeling. In contrast, MSE-derived indices may be able to capture subtle, multiscale irregularities in the MAW signal as well as the local variability and long-range correlations, hence providing a more comprehensive view and richer quantification of signal complexity and the underlying physiological variability. The physiological relevance of this multiscale perspective is supported by previous works in the field, i.e., Cerutti et al. [59] observed an increase in the entropy of the tachogram during AF episodes, evidencing the increase in irregularity and decrease in predictability of the time-series. This increase in tachogram’s irregularity is mainly related to the impossibility of identifying the two classical low frequency and high frequency rhythms present in the tachogram during normal SR episodes. Furthermore, Mainardi et al. [60] demonstrated that nonlinear measures of atrial electrograms could differentiate among different degrees of AF episodes, hence providing a direct link between entropy-based measures and arrhythmia severity. Their study highlighted that higher estimates of complexity measures were obtained for time-series with more chaotic AA, which is often related to a greater heterogeneity in conduction pathways and reentrant circuits, which are main characteristics of atrial remodeling.

### Machine learning and classification models

LDA optimal model for each MSE-based approach was found for limb lead II, while KSVM optimal models were mainly found for precordial lead V2. Each MSE-based model was then combined with the traditionally used indices regarding amplitude and frequency characteristics.

The LRS time-scales showed up to be the most significant ones for the models, as nearly all parameters selected correspond to SE computed at $\tau \ge$ 20. The most relevant standard index was found to be the DAF. Models based on DAF and MSE-based characteristics are able to correctly classify more than 90% of the patients into either AF relapse or SR maintenance class, with independence of the MSE approach considered. Particularly, KSVM provides a higher AUC than 96% with Se and Sp values higher than 92%.

Statistical models have been previously used in [4, 5, 54] but with limited AUC results, mainly between 60 and 70% or limited Se and Sp values. Higher AUC values can be found in the multivariate model proposed by [26] reaching a maximum of 86% correct classification, still lower than the results presented here for the MSE LDA classification model. The LDA model in [26] considers mainly autonomic regulation measures while only incorporating symbolic dynamics as the nonlinear complexity approach, while here the main core of the models relies on the use of nonlinear dynamics. This suggests that entropy-based complexity measures, even from brief ECG segments, may capture clinical temporal dynamics of AA more effectively than traditional autonomic markers. It is also worth noticing that while in [26] 2-h Holter segments were analyzed, this work only needs of 1.5 min ECG segment prior to ECV shock, which makes it a more feasible approach in a clinical setting.

## Conclusion

Based on the actual findings, it could be concluded that ECV outcome prediction based on standard characteristics such as the DAF, FWA, or SE provides better predictive accuracy when computed over the more accessible limb leads. FWAn presented the best discriminant abilities in lead V3, thus indicating the importance of the AA extraction algorithm and spatial information, even though a deeper study needs to be performed.

MSE-based indices outperformed standard characteristics in terms of AUC when measured over precordial leads, particularly when long-range time-scales are considered, thus suggesting that AF triggering processes consist of long-range correlations with slow dynamics which can be better characterized at larger time-scales. Aggregate measures of MSE-based indices, particularly the RMSE $\mathcal {S_{LRS}}$, improve ECV outcome prediction in more than 5% when compared to standard or single scale measures in terms of AUC. Hence, suggesting the idea that entropy rate variations are able to characterize the AF triggering and/or maintenance processes that occur in the atria.

Finally, statistical models combining MSE-based indices and DAF provide better discrimination between AF relapse and SR maintenance groups in ECV outcome prediction, outperforming both standard and complexity indices in more than 3% and up to nearly 30%.

This work, although providing a full and extensive characterization of three different approaches of multiscale entropy over the 12-lead ECG, still presents some limitations. A broader database should be considered; in this way, a better and more representative characterization of the proposed parameters would be obtained. Hence, considering further validation using alternative cross-validation schemes over these larger databases will be essential to establish the clinical reliability of the model. The AA extraction algorithm should be selected based on minimal residue, as standard *f*-waves amplitude characterization is highly influenced by it. Finally, a deeper study based on a possible coupled analysis of leads reflecting both left and right atrium AA activity should be considered along with the inclusion of demographic and clinical variables that could add valuable information to the model.

Furthermore, the incorporation of alternative entropy kernels within the MSE entropy framework, including linear and model-based approaches along with other complexity measures, should be investigated as they could provide further insight into the mechanisms underlying AA, potentially contributing to the refinement of individualized risk stratification strategies.

## Declarations

### Disclaimer

The funders had no role in the design of the study; in the collection, analyses, or interpretation of data; in the writing of the manuscript; or in the decision to publish the results.
